# Emergency shelter allocation planning technology for large-scale evacuation based on quantum genetic algorithm

**DOI:** 10.3389/fpubh.2022.1098675

**Published:** 2023-01-06

**Authors:** Yang Yin, Xiangcheng Zhao, Wei Lv

**Affiliations:** ^1^School of Management, Wuhan University of Technology, Wuhan, China; ^2^School of Safety Science and Emergency Management, Wuhan University of Technology, Wuhan, China; ^3^Shandong Hi-Speed Urban & Rural Development Group Co., Ltd., Jinan, China

**Keywords:** urban safety, emergency shelter, allocation planning, evacuation, quantum genetic algorithm

## Abstract

**Introduction:**

Shelter allocation is one of the most important measures in urban disaster prevention and mitigation planning. Meanwhile, it is essentially a comprehensive planning problem combining resource allocation and traffic routing. A reasonable allocation scheme can avoid congestion, improve evacuation efficiency, and reduce the casualty rate. Owing to the large region and large evacuation population demand, quickly solving the complex allocation problem is somewhat challenging, and thus, the optimal results are difficult to obtain with the increase of evacuation scale by traditional allocation methods.

**Methods:**

This article aims to establish a shelter allocation model for large-scale evacuation, which employs an improved quantum genetic algorithm (IQGA) based on spreading operation and considering the total evacuation distance, the capacity constraint of evacuation sites, and the dispersion of allocation results, and compare allocation schemes of the spreading model with those of models that consider different constraints.

**Results and discussion:**

Results show that the allocation model with the spreading operation has better allocation results than that without the spreading operation. For the allocation model with spreading operation, the spreading model with different spreading speeds is more reasonable than that with the same spreading speed, and the allocation results are closer to the ideal results with the increase of constraints. In addition, according to the allocation results, the evacuation route map and the evacuation heat map are drawn to intuitively understand the distribution scheme of each shelter.

## 1. Introduction

Many areas of the world are vulnerable to disasters, threatening the lives of urban residents and causing major property damage to the country (1). These disasters include natural disasters, such as the 2011 earthquake in Japan, the super typhoon “Lekima”, and the Indian Ocean tsunami, and man-made disasters, such as the explosion accident in Tianjin Binhai New Area and the Chernobyl nuclear disaster. Emergency shelters are ordinarily established as disaster prevention and mitigation facilities in economically developed urban areas, they are the evacuation areas that provide safe assembly spaces and essential for the rescue of the wounded (2, 3). Therefore, when disasters or accidents occur, there would be tremendous amounts of urban residents who need quick allocation of shelters. In this context, developing models for optimizing urban emergency shelter planning is significant for urban disaster prevention and mitigation and people's life safety protection (4). Location selection, evacuation route planning, and shelter allocation are the most crucial issues correlated to emergency shelter planning. Therefore, in recent years, numerous studies have emerged on the modeling or methods for shelter location selection, evacuation route planning, and shelter allocation.

Regarding shelter location selection models, in early research, most of the studies on humanitarian emergency facility location problems prefer to apply mathematical programming methods such as P-Center (5), P-Median (6), and Max covering approach (7, 8). But, in this way, models are built to consider a single factor, while realistic situations are far more complicated. Therefore, some studies considering multiple factors or multiple methods have begun to emerge. Trivedi and Singh (9) presented a hybrid algorithm for shelter location selection by proposing a hybrid multi-objective decision model based on the analytic hierarchy process (AHP), fuzzy theory, and goal programming approach; the model considered evacuation demand, shelters utilization, budgetary cost, and subjective vagueness of decision-makers and finally explored the applicability of theories of multi-criteria decision-making support in emergency shelter location selection. Xu et al. (10) put forward seven principles for shelter location selection, constructed a multi-objective location model and program with these principles, extended the P-Median model and location set-covering problem model, and finally selected the city of Yangzhou to verify this model and location program. Song et al. (11) developed an integrated method by combining the qualitative flexible multiple criteria (QUALIFLEX) method and the rough set theory to solve the sustainable shelter location selection problem, and the Wenchuan county was employed to verify the efficiency and practicability of the method.

Regarding evacuation route planning methods, the mainstream research methods include cellular automata modeling (12, 13), agent-based modeling (14, 15), and mathematical optimization methods such as linear programming methods (16, 17) and intelligence/ heuristic algorithms (18–21). The cellular automata and agent-based models could reflect human psychology and behavior, be closer to the real situation, and present more accurate results, while the modeling process is usually complex. The optimization methods could employ a series of mathematical models to replace complex practical problems and then produce optimal solutions through tools or methods such as intelligent algorithms, CPLEX, and LINGO. Compared to the modeling approaches, optimization methods could produce optimal solutions for evacuation route planning with a short solving time.

Regarding shelter allocation models, there are two main categories, namely, the Euclidean distance model and the Network distance model. Buffer or radius methods (22), ordinary Voronoi diagram (OVD), and weighted Voronoi diagram (WVD) (23, 24) are three typical methods that adopt Euclidean distance. They employ straight-line distances to produce allocation solutions for shelters and thus cannot represent the actual evacuation routes. As for network distance, some scholars incline to employ ArcGIS network analysis tools (25, 26) to produce allocation solutions, which are closer to the real evacuation route but ignore the capacity limit of shelters and thus may cause too many people to evacuate toward the same shelter. Other scholars also adopt integration methods to consider shelter capacity. Jiang et al. (27) established a hierarchical UHM network based on central place theory, and the shelter allocation is solved by a series of geographic information system technologies. Li et al. (28) designed a two-stage mathematical programming model for shelter allocation; in the second-stage model, the shelter allocation was solved by minimizing the total evacuation distance and considering the capacity of the shelter. Li et al. (29) developed an algorithm for shelter allocation, in which a shift insertion mechanism to reduce total evacuation distance and to improve the spatial continuity of allocation results was employed, taking into account the capacity constraints of the shelter. It is worth noting that most studies prefer to apply adjacent communities or streets as evacuation demand regions, less investigate discrete regions like buildings. From the macroscopic view, the shelter allocation problem can be treated as the knapsack problem, which is known as an NP-complete problem. To solve the problem, intelligent algorithms such as the genetic algorithm (30), tabu search algorithm (31), and particle swarm optimization algorithm (32) have been applied in some studies. However, the high computational complexity would cause these algorithms to fall into local optimum easily if they were not improved before solving the large-scale scenario problem.

The purpose of this study is to develop a realistic and objective model to solve the shelter allocation problem quickly and effectively. Therefore, an improved spreading quantum genetic algorithm (ISQGA) considering discrete regions and capacity constraints is proposed and elaborated in detail with case application. It is hoped that the method could provide an effective and quick planning scheme for the emergency commander and urban managers. This article is arranged as follows. In Section 2, a shelter allocation model considering discrete regions and capacity constraints is established. In Section 3, the improved algorithm for solving the model is explained. In Section 4, a case application is presented and discussed. Finally, in Section 5, the whole study is summarized and concluded.

## 2. Shelter allocation model development

### 2.1. Shelter allocation problem

The shelter allocation problem can be considered a planning problem. Suppose there are some residential buildings and several shelters in a certain city space, the task of allocation is to plan the evacuation destination shelter for each building and the evacuation paths in the road network. In the study, capacity limitation of shelters, continuity and rationality of results, and the shortest total evacuation distance are also needed to be considered in the problem.

### 2.2. Shelter allocation model

Before developing the allocation model, specifying the factors or variables related to the problem is necessary, and Table 1 shows the definitions for sets, indexes, and variables that would be used in the model.

**Table 1:** Definitions for sets, indexes, and variables of the problem.

**Type**	**Symbol**	**Definition**
Sets	*BS*	The set of buildings within the study area
	*SS*	The set of emergency shelters
Indexes	*i,a*	The *i*^th^ or *a*^th^ shelter, i,a ∈ BS
	*j,k*	The *j*^th^ or *k*^th^ building, j,k ∈ BS
	*tn*	The number of buildings within the study area
Variables	*DC*	The constraint value of discrete distance
	*SC*	The constraint value of spreading initial points
	*CC_*i*_*	The capacity of the *i*^th^ shelter
	*SI_*i*_*	The distance of spreading initial point of the *i*^th^ shelter
	*BD_*jk*_*	Distance between the *j*^th^ building and the *k*^th^ building
	*D_*ij*_*	The distance between *i*^th^ buildings and *j*^th^ shelter
	*X_*ij*_*	1, if the *j*^th^ building is assigned to the *i*^th^ shelter; 0, else
	*Y_*ij*_*	1, if *BD_*jk*_*≥ DC; 0, else
	*BP_*j*_*	The population within the *j*^th^ building
	*TP*	The total population within study area

In this model, shortening the evacuation routes and keeping contiguity are two important objectives. To minimize the total distance of all evacuation routes from each building to its assigned shelter, the first objective function *F*_1_ can be set up as follows:


(1) $$\begin{array}{l} \min F_{1}=\underset{i\in SS}{\sum }\underset{j\in BS}{\sum }D_{ij}X_{ij} \end{array}$$

**Figure 1:**
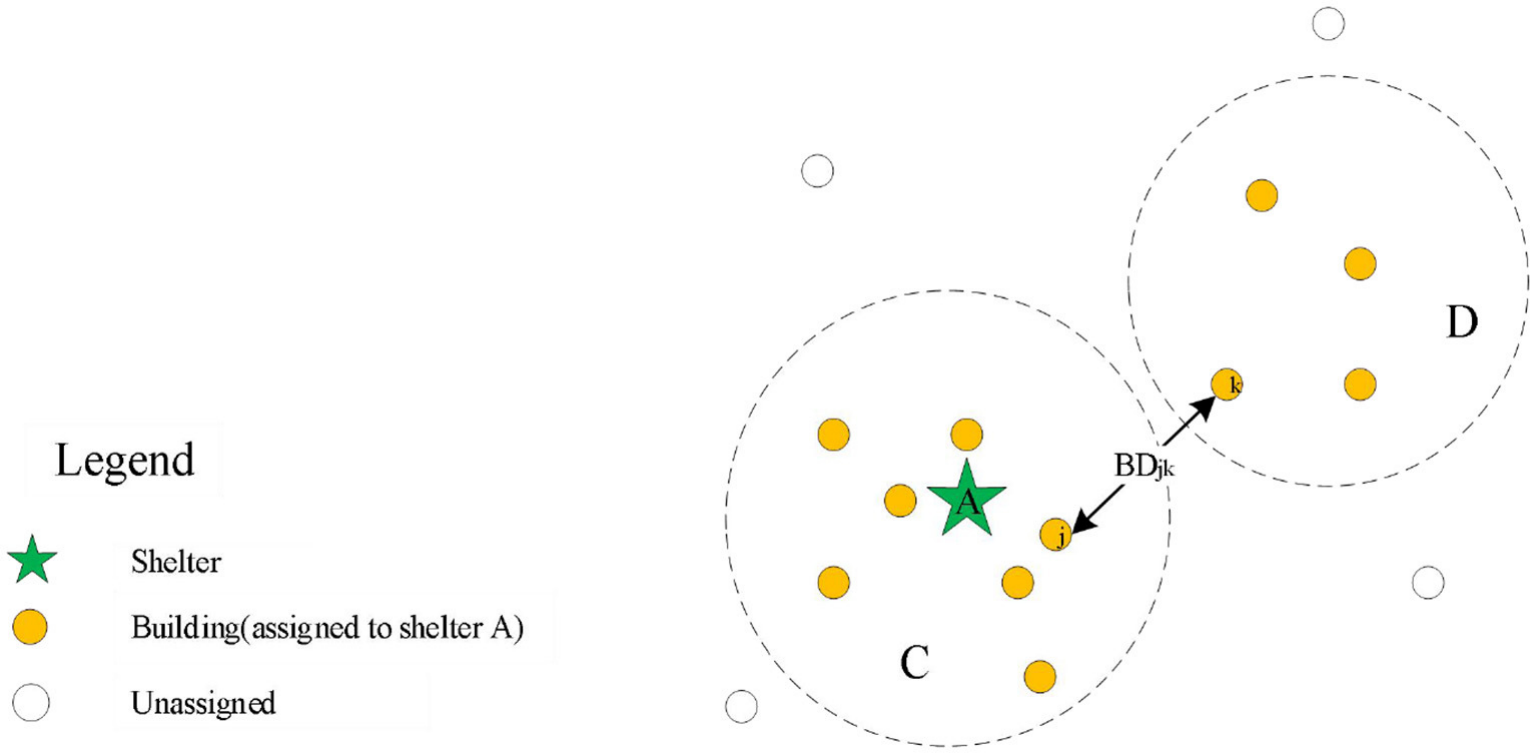
The scenario analysis of discrete allocation result.

In the planning of shelter allocation, if we only consider the factor of total evacuation distance, the distance between the two buildings assigned to the same shelter may be too large in the allocation results, as shown in Figure 1, which would induce a high probability of congestion during evacuation. Therefore, to avoid the generation of congestion in the evacuation process, the continuity of the allocation is also necessary. Considering the buildings are not connected to each other in the discrete layout, we introduce a discrete penalty function *F*_2_ to reduce the occurrence of discrete points:


(2) $$\begin{array}{lll} \min F_{2} & = & \underset{i\in SS}{\sum }\underset{j,k\in BS}{\sum }BD_{jk}X_{ij}Y_{jk}\rho^{2} \end{array}$$

(3) $$\begin{array}{lll} \rho & = & floor\left[\frac{BD_{jk}-DC}{DC}\right] \end{array}$$

**Figure 2:**
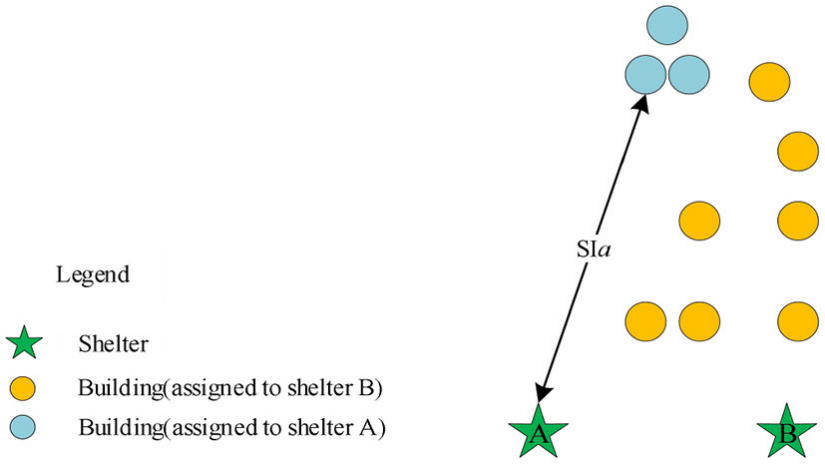
The scenario analysis of the spread initial point.

Considering the capacity of the shelters located within a certain city space may not be able to meet the need of the residential buildings, some other shelters located outside the space should be introduced into the scope of shelters for allocation. Compared with internal shelters, each external shelter has a longer useless distance to find the first service object. When using the spread method proposed in this study, if the location of the initial point is not constrained, the distance between the initial point and its corresponding shelter may be too large when the total distance is the same, as shown in Figure 2.

Therefore, we introduce a penalty function of spread initial point *F*_3_ to reduce the occurrence of unreasonable starting points in Figure 2:


(4) $$\begin{array}{lll} \min F_{3} & = & \underset{a\in SS}{\sum }SI_{a}\mu^{2} \end{array}$$

(5) $$\begin{array}{lll} \mu & = & floor\left[\frac{SI_{a}-SC}{SC}\right] \end{array}$$

Combining the three objective functions, as well as considering the capacity limitation of shelters, we propose the following model to solve the shelter allocation problem:


(6) $$\begin{array}{l} \min F=F_{1}+F_{2}+F_{3}\\ \;s.t. \end{array}$$

(7) $$\begin{array}{lll} \underset{j\in BS}{\sum }X_{ij}BP_{j} & \leq & CC_{i}\;\forall i\in SS \end{array}$$

(8) $$\begin{array}{lll} \underset{j\in BS}{\sum }X_{ij} & = & \text{1,}\forall i\in SS \end{array}$$

(9) $$\begin{array}{lll} X_{ij} & = & \left\{0,1\right\},\forall i\in SS\forall i\in BS \end{array}$$

Formula (6) is the total objective function, which is employed to minimize the total evacuation distance and improve the continuity and rationality of the allocation results. Formula (7) indicates that the number of people allocated to each shelter cannot exceed its capacity limit. Formula (8) means that each building can only be allocated to a single shelter. Formula (9) produces the binary constraints.

In addition, considering the difficulty of obtaining the population data of each building, in this study, we adopt a strategy of randomly generating the population for each building by the following formula:


(10) $$\begin{array}{lll} EBP_{j} & = & FP+RP_{j}\;j\in BS \end{array}$$

(11) $$\begin{array}{lll} FP & = & round\left(\frac{TP}{2tn}\right) \end{array}$$

(12) $$\begin{array}{lll} RP_{j} & = & randi\;j\in BS \end{array}$$

(13) $$\begin{array}{lll} TP & - & FP\cdot tn=\underset{j\in BS}{\sum }RP_{j} \end{array}$$

Formula (10) is the random population of each building, which is composed of two variables, namely, fixed population (*FP*) and random population (*RP*_i_). For population data in each building, it is difficult to get them directly from the statistical department, but it is obviously not realistic to set the same population in all the buildings. Therefore, a randomness method for generating the number of populations in each building is proposed. Half of the population in the area was evenly distributed to each building, and the other half was randomly assigned to each building. Although this scheme may not fully describe reality, it does reflect the characteristics of the randomness distribution of the real population. Formula (11) shows the fixed population, which is obtained by rounding the ratio of half of the total population to the number of buildings. Formulas (12) and (13) represent the generation method of the random population, which is obtained by randomly assigning the remaining number of people to each building. In addition, for *FP*, *RP*_*j*_, and *EBP*_*j*_, their values are all integers.

## 3. Shelter allocation algorithm

### 3.1. The spread principle for the emergency shelter allocation model

The spread principle proposed in this study is relatively simple, just as water flows in the pipeline; we regard each shelter as the source of water and the road network as a pipeline. If each shelter is given a spread speed S, the shelter will spread along the road to find the service objects, and whenever the distance of the outward spread reaches S, the expansion is paused and all the buildings within [SR,SR-S] range are recorded, where SR represents the distance between the pause location and its belonging shelter, and then the evacuation destination of these buildings is analyzed based on the distance. The shelters continue to spread out in this way until all buildings are assigned to the shelter after the allocation. To be clarified, the spread process proceeds along the actual road network, so that the boundary of each pause is not a standard circle, but possibly an irregular regional boundary, where the speed S actually specifies the outward spread distance of each step of the operation.

**Figure 3:**
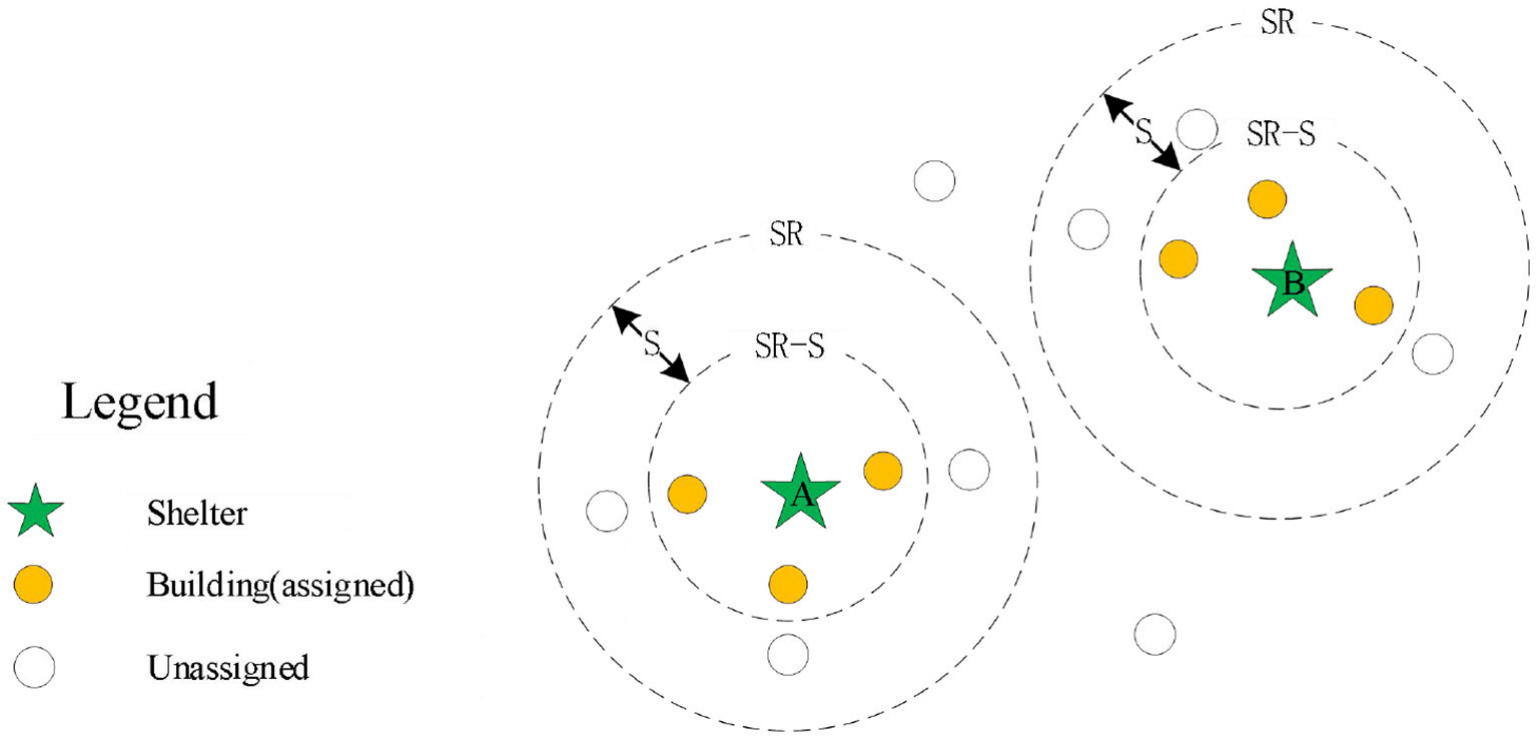
The scenario analysis of without intersecting area.

The following situations may be encountered during the spread process:

In *situation 1*, as shown in Figure 3, there is no intersection region between the spread ring of the two shelters, that is, there are no duplicate buildings within the current spreading of the two shelters. Therefore, we only need to consider the capacity constraints of shelters for the allocation of buildings within the spread ring to the shelter where the spread ring belongs. First, in order to keep continuity, we sequence the buildings within the spread ring from small to large according to the distance and then assign the buildings one by one to the designated shelter according to this sequence, until the maximum capacity of the shelters is reached or all buildings have been allocated. When the maximum capacity of the shelters is reached, the shelters stop spreading and then remove these shelters from the set U (the set of shelters with remaining capacity). If there are any shelters in the set U, they will continue to spread outward; otherwise, the spreading operation will be ended and then the results of shelter allocation will be analyzed.

**Figure 4:**
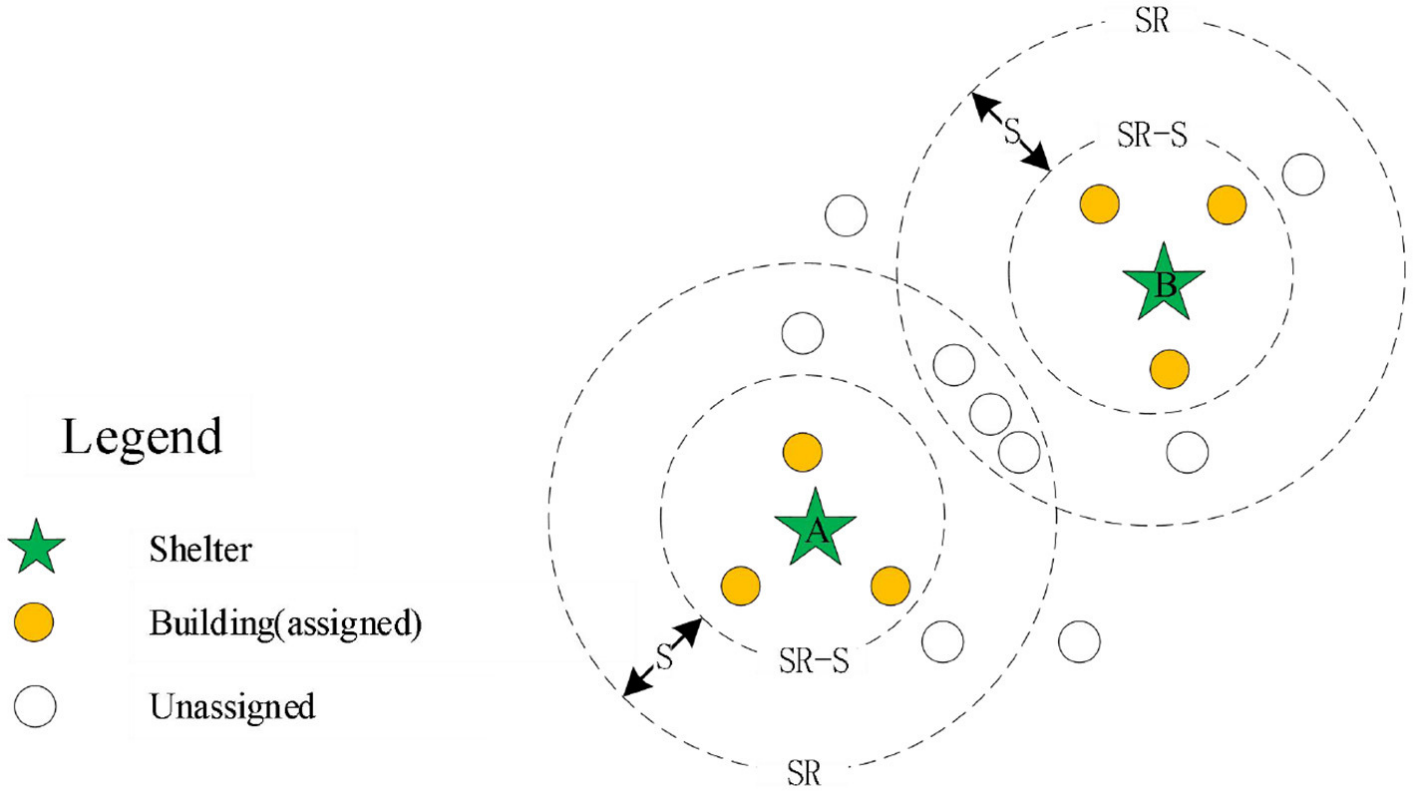
The scenario analysis of with intersecting area.

In *situation 2*, as shown in Figure 4, there is an intersection region between the spread ring of the two shelters, that is, there are some duplicate buildings within the current spreading of the two shelters. Therefore, we need to solve the assigned problem of duplicate buildings in the intersection region. First, we pre-assign the duplicate buildings to the shelter according to the distance, that is, if the building is close to the shelter A, it will be pre-assigned to the shelter A. If the building is close to the shelter B, it will be assigned to the shelter B. Based on this principle, all duplicate buildings in the intersection region are pre-allocated. Then, the buildings pre-allocated are merged into the shelter A, and the buildings that do not intersect with other shelters are into the same set MG. Finally, the buildings in the set MG are assigned according to the allocation method of Situation 1. In addition, for the end condition of the spreading operation, when there are no shelters in the set U or all buildings within the study region are allocated, it is regarded as the end of the spreading operation.

### 3.2. Improved quantum genetic algorithm (IQGA)

The quantum genetic algorithm (QGA) was first proposed by Narayanan and Moore in 1996 (33), which is a newly developed probability evolution algorithm combining the genetic algorithm and quantum computing, and it introduces qubit as the minimal unit of the chromosome. To a certain extent, traditional genetic algorithm (GA) easily falls into local maxima, and slow convergence speed and poor accuracy of search results are overcome. The QGA could very well overcome these deficiencies and therefore has been widely used in various optimization problems in the last 10 years. A conventional QGA mainly includes quantum bit coding, chromosome measurement, fitness calculation, and evolutionary operation based on a quantum rotating gate (34). Therefore, qubit coding and quantum rotating gate are two key elements to distinguish quantum genetic algorithm and traditional genetic algorithm.

#### 3.2.1. Quantum bit coding

**Figure 5:**
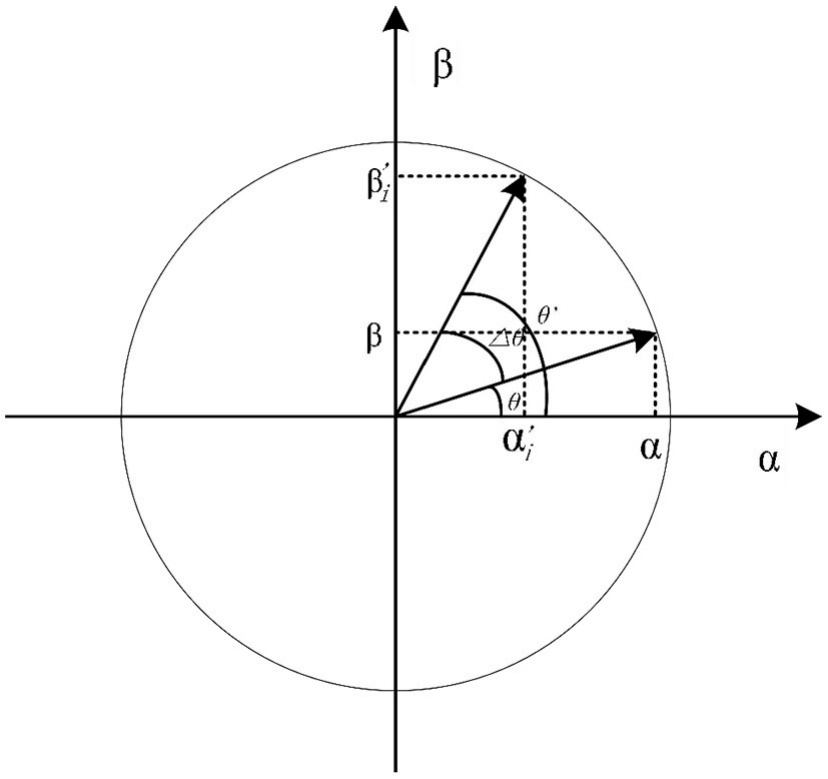
The polar plot of qubit state in QGA.

In quantum theory, there is a fundamental property that a qubit can be expressed as a linear superposition of |0〉 and |1〉, as shown in Figure 5 (35). Its formula is expressed as follows:


(14) |φ〉 =α|0〉+β|1〉

(15) $$\begin{array}{lll} {\left|\alpha \right|}^{2} & + & {\left|\beta \right|}^{2}=1 \end{array}$$

where α and β are complex numbers that specify the probability amplitudes of the corresponding states. |α|^2^ and |β|^2^ are probabilities that the qubit will be translated into a ‘0' state and ‘1' state, respectively. Formula (15) represents the normalization condition in which α and β satisfy.

For example, a quantum chromosome with four qubits can be shown as follows:


(16) $$\begin{array}{l} q=\left[\begin{array}{c} \alpha_{1}\\ \beta_{1} \end{array}\begin{array}{c} \alpha_{2}\\ \beta_{2} \end{array}\begin{array}{c} \alpha_{3}\\ \beta_{3} \end{array}\begin{array}{c} \alpha_{4}\\ \beta_{4} \end{array}\right]=\left[\begin{array}{c} \frac{1}{\sqrt{2}}\\ -\frac{1}{\sqrt{2}} \end{array}\begin{array}{c} -\frac{1}{\sqrt{3}}\\ \frac{1}{2} \end{array}\begin{array}{c} \frac{\sqrt{3}}{2}\\ \frac{1}{2} \end{array}\begin{array}{c} -\frac{\sqrt{3}}{2}\\ -\frac{1}{\sqrt{3}} \end{array}\right] \end{array}$$

For Formula (16), the probability of each qubit converting to a ‘0' state and a ‘1' state is shown in Table 2.

**Table 2:** The probability table for producing the state of each qubit.

**Sate**	**First qubit**	**Second qubit**	**Third qubit**	**Fourth qubit**
0	1/2	1/3	3/4	3/4
1	1/2	1/4	1/4	1/3

Therefore, the four-qubits quantum chromosome will take the states of |0000〉, |0100〉, |0010〉, |0001〉, |1000〉, |1100〉, |1010〉, |1001〉, |0110〉, |0101〉, |0011〉, |1110〉, |1011〉, |0111〉, |1101〉, and |1111〉, in the probabilities of 3/32, 9/128, 1/32, 1/24, 3/32, 9/128, 1/32, 1/24, 3/128, 1/32, 1/72, 3/128, 1/72, 1/96, 1/32, and 1/96, respectively. Thus, the state of the quantum chromosome can be expressed as follows:


(17) $$\begin{array}{l} |\varphi_{q}\rangle \;=\frac{3}{32}|0000\rangle +\frac{9}{128}|0100\rangle +\frac{1}{32}|0010\rangle +\frac{1}{24}|0001\rangle \\ \;+\frac{3}{32}|1000\rangle +...\frac{9}{128}|1100\rangle +\frac{1}{32}|1010\rangle +\frac{1}{24}|1001\rangle \\ \;+\frac{3}{128}|0110\rangle +\frac{1}{32}|0101\rangle +\frac{1}{72}|0011\rangle +...\frac{3}{128}|1110\rangle \\ \;+\frac{1}{72}|1011\rangle +\frac{1}{96}|0111\rangle +\frac{1}{32}|1101\rangle +\frac{1}{96}|1111\rangle \end{array}$$

#### 3.2.2. Improved rotation gate

Compared with the genetic algorithm (GA), QGA employs a rotation gate to replace the variation operator of GA to guide the evolution of chromosomes. For the rotation gate of QGA, it is used to convert the qubit state of the current chromosome to the corresponding qubit state of the chromosome with the best fitness. In addition, with the rotation gate, QGA has a greater possibility than GA to obtain the global optimum, since quantum chromosomes have various linear combinations transformed into binary (36).

According to Liu et al. (37), the conventional rotation gate operation is achieved through a fixed rotation angle θ, as shown in Figure 5. To enhance the computational efficiency of rotation gate operation, and as far as possible avoid that fall into the local optimum due to the angle selection being too small, or missing the global optimum due to the angle selection being too large, an improved rotation gate is proposed based on Formulas (18)-(22). It modifies the angle θ by a dynamic updating operation. The formula for qubit dynamic updating is given as:


(18) $$\begin{array}{lll} \text{Δ}\theta & = & \theta_{\min}+f^{{}^{\prime }}*\left(\theta_{\max}-\theta_{\min}\right) \end{array}$$

(19) $$\begin{array}{lll} f^{{}^{\prime }} & = & \frac{f_{x}-f_{best}}{f_{x}} \end{array}$$

(20) $$S\left(\theta \right)=sign(\text{Δ}\theta )=\left\{ \begin{array}{l} 1\;s_{best}>s\\ -1\;s_{best}<s\\ 0\;s_{best}=s \end{array}\right.$$

(21) $$\begin{array}{lll} \theta^{{}^{\prime }} & = & S\left(\theta \right)*\text{Δ}\theta \end{array}$$

(22) $$\begin{array}{l} \left[\begin{array}{l} \alpha_{\prime }^{i}\\ \beta_{\prime }^{i} \end{array}\right]\;=\;U\left(\theta^{\prime }\right)\;\left[\begin{array}{l} \alpha_{i}\\ \beta_{i} \end{array}\right]\\ \;=\;\left[\begin{array}{l} \cos\left(\theta^{\prime }\right)\;-\;\sin\left(\theta^{\prime }\right)\\ \sin\left(\theta^{\prime }\right)\;\cos\left(\theta^{\prime }\right) \end{array}\right]\;\left[\begin{array}{l} \alpha_{i}\\ \beta_{i} \end{array}\right] \end{array}$$

where θ′ is the qubit angle to update chromosome, *S* is the qubit individual state of current fitness, *S*_*best*_ is the qubit individual state of the optimum fitness, ${\left[\alpha_{i}\beta_{i}\right]}^{T}$and ${\left[{\alpha^{\prime }}_{i}{\beta^{\prime }}_{i}\right]}^{T}$are the probability amplitude before and after the *i*^*th*^ qubit update, Δθ is the rotation angle, and *S*(θ) is the direction of quantum gate rotation, *f*_*x*_ is the fitness value of current chromosome, *f*_*best*_ is the global optimal fitness value of records, *U*(θ′) is the adjustment operation of quantum rotation gate, and θ_min_(θ_max_) is the minimum (maximum) value of the given rotation angle range, which is given in the next paragraph.

The value of the rotation angle range [θ_min_, θ_max_] has a significant influence on searching the global optimum, which determines the value of Δθ the larger θ_max_ is, the larger Δθ is, the smaller θ_min_ is, and the smaller Δθ is. A large rotation angle Δθ may lead to missing the global optimum; conversely, too small Δθ may lead to slow convergence and cannot jump out of the local optimum. We usually set the value Δθ to be between 0.001π and 0.05π (38).

#### 3.2.3. Algorithm flow based on spread operation

For the spread strategy, it is necessary to give an initial speed for shelters to spread outward. Therefore, in this study, the spreading speed of the shelter is taken as a variable, and the IQGA is used to solve the optimal spreading speed; using the objective function as a fitness function of IQGA satisfies the constraints of Formulas (7)-(9). In addition, there are two ways to employ spreading speed as a variable: each refuge uses the same diffusion velocity, and thus, there is only one variable; each shelter uses a different spreading speed. Therefore, the number of variables is equal to the number of shelters, and we will compare the results of the two ways in the later context.

The concise flow of the IQGA considering spread operation can be summarized as follows:

(1) Initialize population *Q*(t) and randomly generate *n* quantum chromosomes.
(2) Measure each individual quantum chromosome in the *Q*(t) and obtain the same spreading speed S or a different spreading speed *S*_i_.
(3) Employ *S* or *S*_i_ to obtain fitness. Take *S* or *S*_i_ as the spreading speed and then obtain the objective function, namely, the fitness value, according to the diffusion result.
(4) Record the optimal spreading speed and its corresponding fitness value.
(5) New population *Q*(t+1) is obtained by employing a quantum rotation gate to update the quantum chromosome.

**Figure 6:**
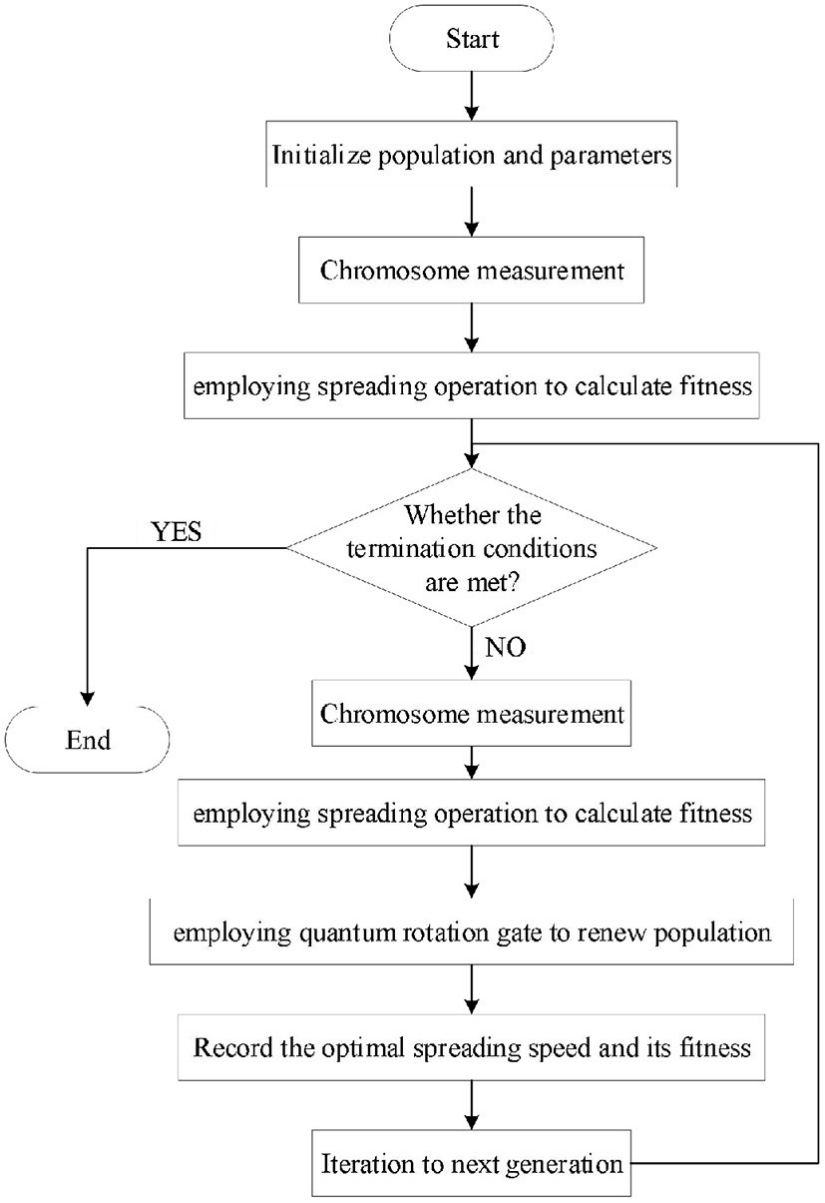
The flow chart of IQGA, considering a spread operation.

The corresponding algorithm flow chart is shown in Figure 6.

## 4. Case application

### 4.1. Study region selection

**Figure 7:**
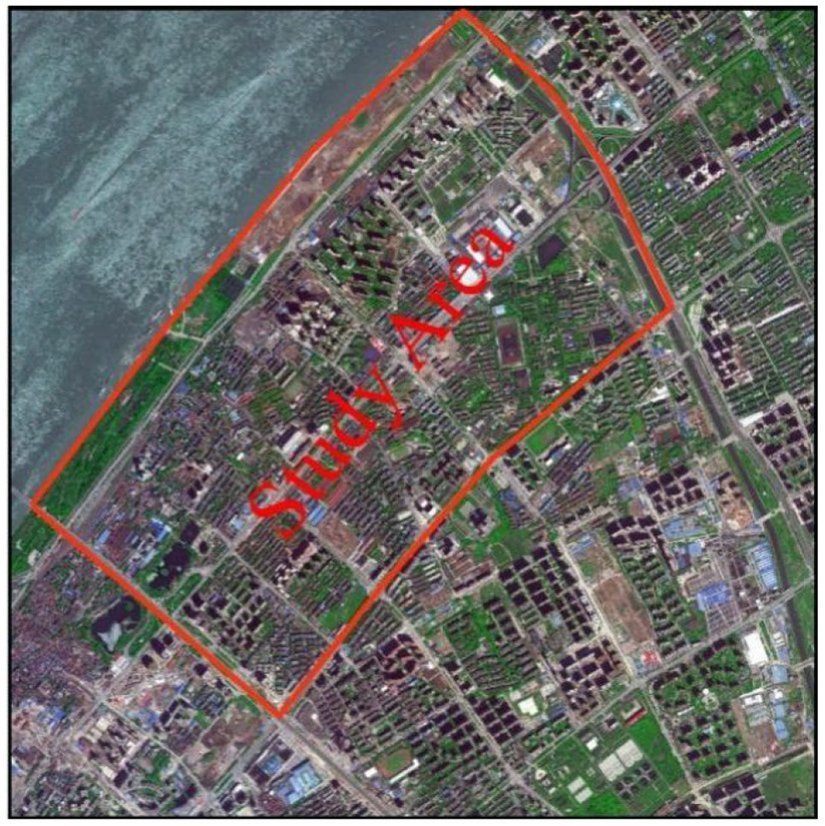
The Yangyuan Street area in the satellite image.

Wuhan, the megacity and the central city in China, is located in the middle reach of the Yangtze River. In this study, a central urban region with a dense population in Wuhan, i.e., the Yangyuan Street region, was selected to study, as shown in Figure 7, which covers an area of 5.24 square kilometers, a large number of chemical plants and a resident population of 109,485.

### 4.2. Data preparation

**Figure 8:**
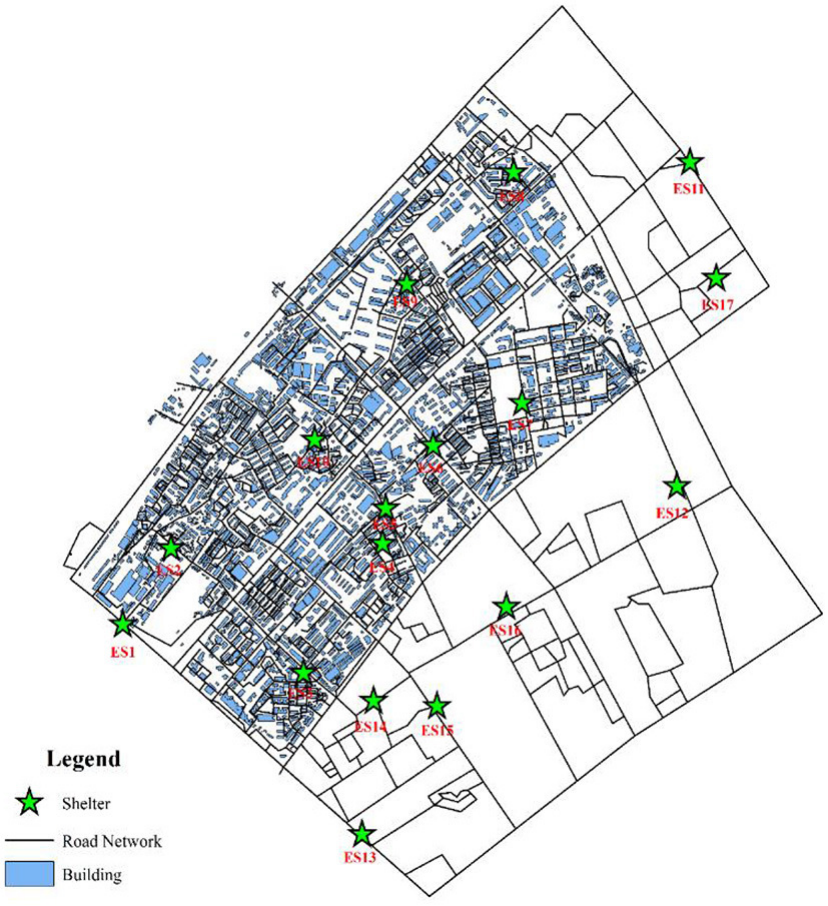
Facilities distribution of the Yangyuan Street region.

The essential data were digitized base on the tile map of Baidu Map, including roads, emergency shelters, and building outlines, as shown in Figure 8.

**Figure 9:**
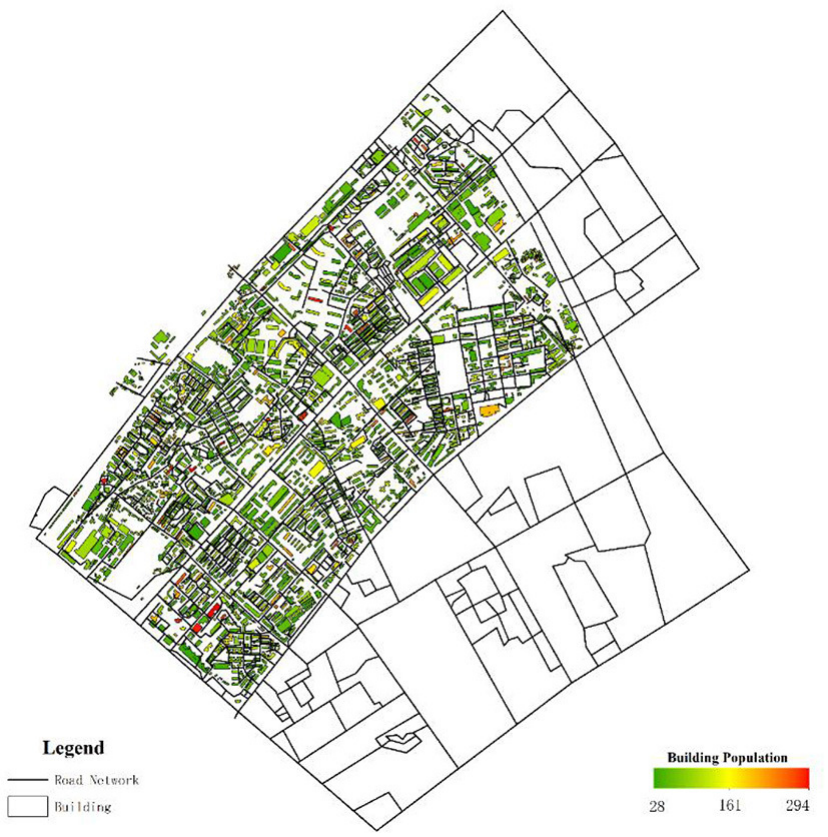
The population distribution of each building.

In addition, when drawing roads outside the study area, we only consider the trunk roads outside the study area as a result of no evacuation demand for the people outside the region. Therefore, the roads outside the study region are much simpler than the roads inside the study region. This study mainly aimed at the temporary shelter of flood disasters for residents by river banks. Therefore, although there are many places in the Yangyuan Street region that can be used as emergency shelters, such as school playgrounds, green parks, and community squares, in order to reduce the computational amount, only large parks and green areas in the region were selected to be temporary emergency shelters. Meanwhile, the shelters adjacent to the Yangtze River were excluded to avoid environmental pollution and flood impact. For the capacity of shelters, the data were obtained from the website introduction and the space measurement of the shelters. In addition, the total capacity of the shelters in the study region was unable to accommodate such a large amount of people, thus several shelters outside the region to meet the evacuation demand of the whole street area were set up. The model parameters and the capacity of each shelter are shown in Tables 3, 4. The model parameters are set based on the general settings of the algorithm. The capacity of the shelter is computed by the ratio of the available area of the shelter to the occupied area per capita, in which the occupied area per capita is 2 m^2^, and the available area is 60% of the land area of the shelter. For discrete distance limit D, we think that when D > 300, the two buildings are discrete and be punished with Formula (2). For the initial point limit L, we think that when L > 1,000, it is punished by Formula (4). In addition, we also randomly assigned the evacuated population to each building through Formulas (10)-(13), as shown in Figure 9.

**Table 3:** Parameter setting value table.

**Parameter**	**Set value**	**Parameter**	**Set value**
Chromosome length *len*	10	Initial point distance *SC*	1,000 m
Iteration times *Gen*	500	Discrete distance *DC*	300 m
Population size *n*	20	Number of buildings *BS*	1,980
Number of shelters *SS*	17		

**Table 4:** The capacity of each shelter.

**Shelter**	**ES 1**	**ES 2**	**ES 3**	**ES 4**	**ES 5**	**ES 6**	**ES 7**	**ES 8**	**ES 9**
Capacity/hm^2^	5.6	0.3	0.6	0.3	0.6	0.3	1.6	0.3	0.3
Shelter	ES 10	ES 11	ES 12	ES 13	ES 14	ES 15	ES 16	ES 17	
Capacity/hm^2^	0.6	7	1.2	1.6	0.3	0.3	0.8	0.6	

### 4.3. Simulation

**Figure 10:**
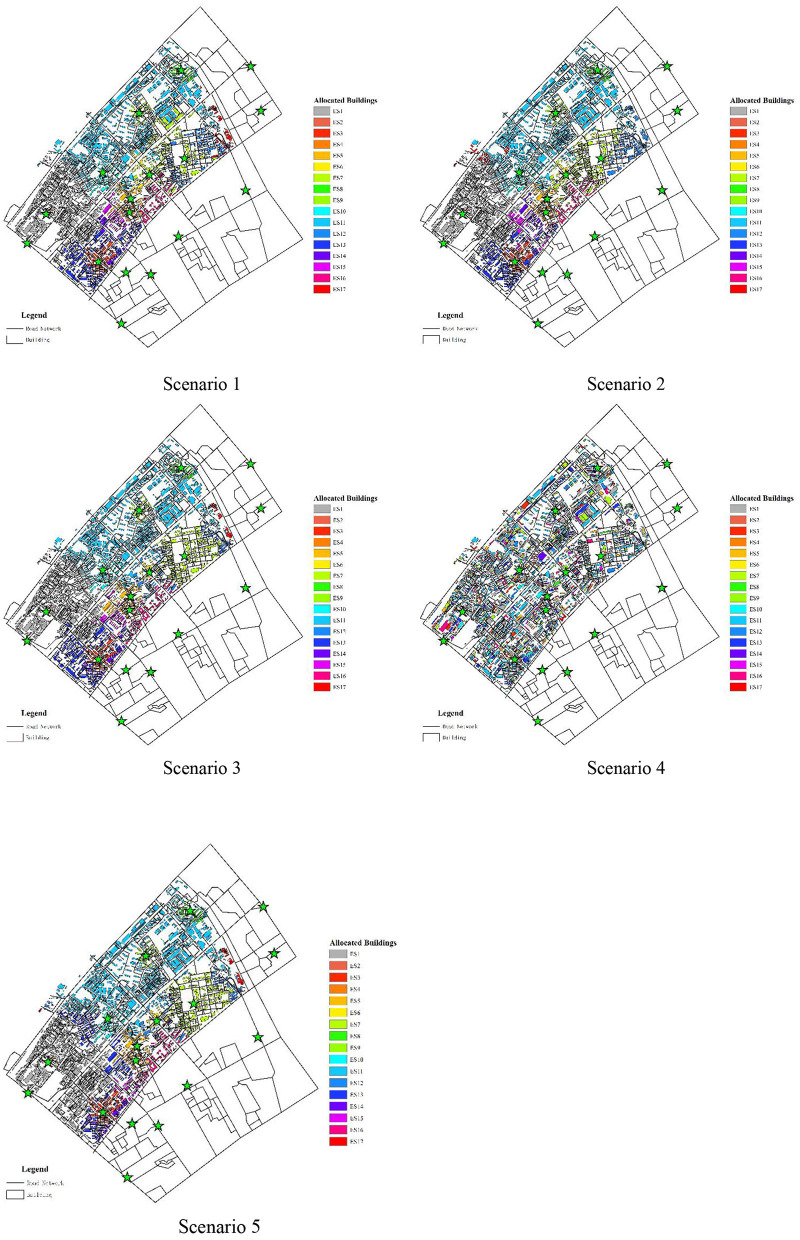
The allocation results of shelters for five scenarios.

In this study, we constructed five scenarios simulation by means of adopting the improved quantum genetic algorithm considering the unchangeable spreading speed *S* and changeable spreading speed *S*_i_. The unchangeable speed means that all shelters are spreading at the same speed, and the changeable speed means that each shelter spreads outward at a different speed. In other words, unchangeable speed means setting only one variable in IQGA, and changeable speed means setting the same number of variables as the number of shelters in IQGA. The five simulation scenarios can be described as follows:

*Scenario 1*, as shown in Figure 10, illustrates the planning of shelter allocation adopting IQGA with changeable spread speed, which considers the total evacuation distance, dispersion, spread initial distance, and capacity constraints.

*Scenario 2*, as shown in Figure 10, illustrates the planning of shelter allocation adopting IQGA with changeable spread speed, which considers the total evacuation distance, discreteness, and capacity constraints.

*Scenario 3*, as shown in Figure 10, illustrates the planning of shelter allocation adopting IQGA with changeable spread speed, which only considers the total evacuation distance and capacity constraints.

*Scenario 4*, as shown in Figure 10, illustrates the planning of shelter allocation adopting IQGA without spreading operation, which considers the total evacuation distance, dispersion, spread initial distance, and capacity constraints.

*Scenario 5*, as shown in Figure 10, illustrates the planning of shelter allocation adopting IQGA with unchangeable spread speed, which considers the total evacuation distance, dispersion, spread initial distance, and capacity constraints.

The corresponding numeric results are listed in Table 5.

**Table 5:** The digitized results for each scenario.

**Scenario**	**Min-fitness**	**Max-distance of spread initial point**	**The number of discrete points**
1	2378,406	1,060.88	36
2	2367,088	3,595.46	30
3	2335,391	936.99	106
4	2.2 E + 07	1,327.24	564
5	1.2 E + 07	778.51	196

**Figure 11:**
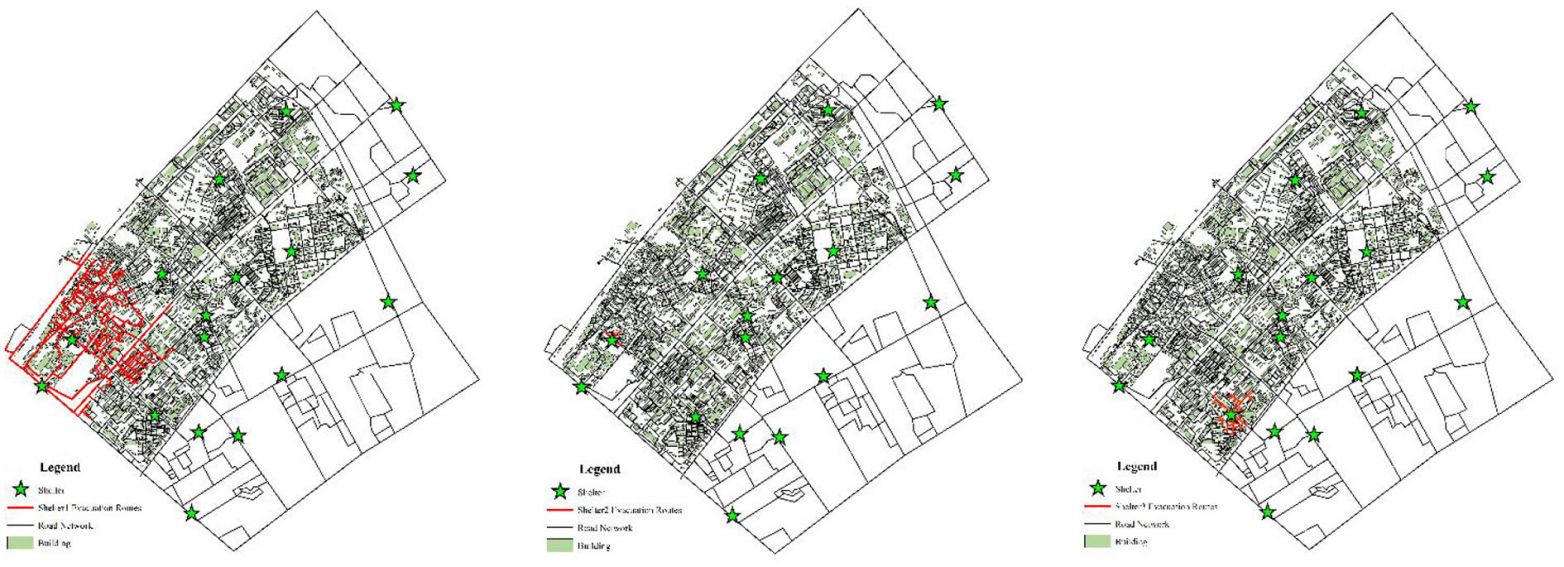
Fitness iteration graph under different scenarios.

As shown in Figure 10, the buildings with the same color represent they are assigned to the same shelter. In terms of color distribution, *Scenario 4* has a chaotic color layout, buildings with the same color are far away, and the discreteness is the highest. Compared with *Scenario 4*, the color layout of other scenes is basically the same, and buildings with the same color are adjacent to each other, so there is a high continuity. To visually compare the allocation results of each scenario, convert the color layout of each scenario in Figure 10 into corresponding numerical results and iteration curves, as shown in Table 5 and Figure 11, and each column data into dimensionless processing, as shown in Table 6. We can observe that *Scenario 4* and *Scenario 5* have the largest fitness values, far exceeding the first three scenarios. The first three scenarios are solved by employing an improved spreading quantum genetic algorithm with changeable speed, and their fitness is not much different. In addition, from the perspective of the number of discrete points, *Scenario 3* has the largest number of discrete points, with stronger discreteness. In terms of the Max-distance of the spread initial point, *Scenario 2* has the largest Max-distance of the spread initial point value, the evacuation completion time will also be greatly extended. Therefore, the results obtained by employing the spreading quantum genetic algorithm considering four factors are more reasonable; as the result, the effectiveness of the method in solving the problem of shelter allocation is proved.

**Table 6:** The digitized results dimensionless processing.

**Scenario**	**Min-fitness**	**Max-distance of spread initial point**	**The number of discrete points**
1	0.0022	0.1002	0.0112
2	0.0016	1	0
3	0	0.0563	0.1423
4	1	0.1948	1
5	0.5171	0	0.3109

### 4.4. Evacuation route and heat map

**Figure 12:**
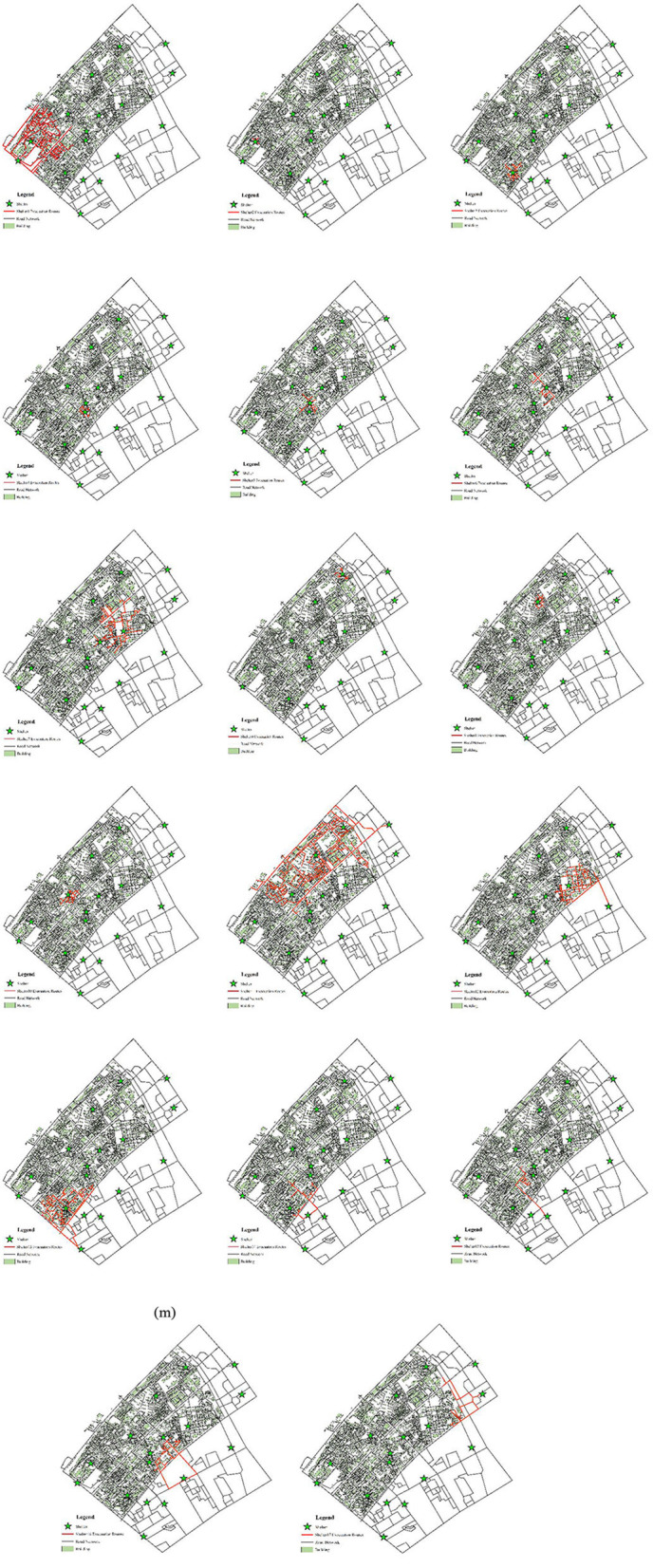
Evacuation routes to each shelter.

**Figure 13:**
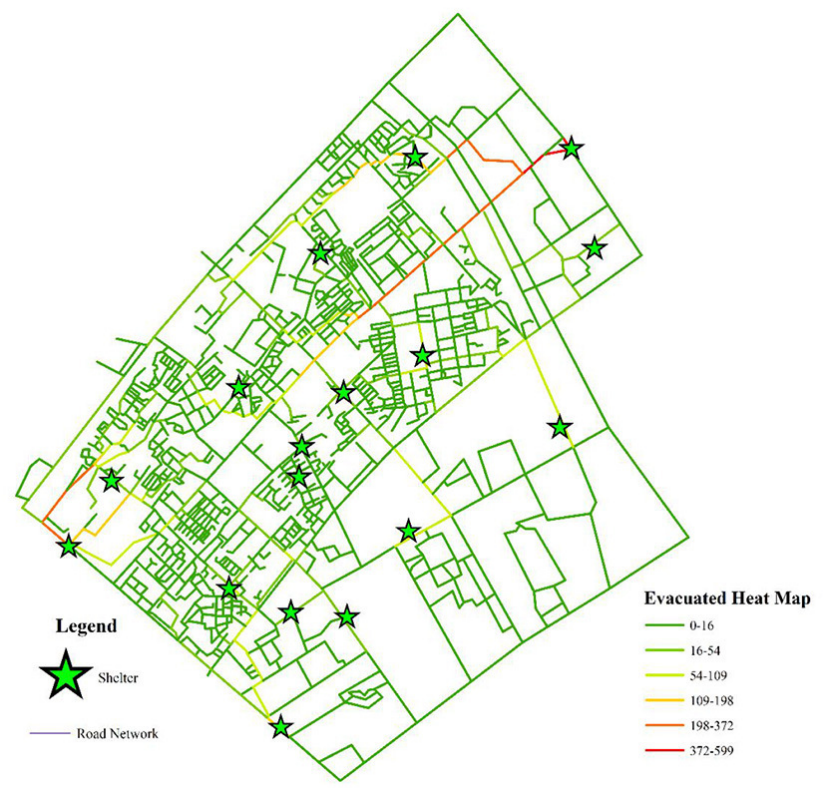
Evacuation heat map of the road network.

Reasonable route planning can effectively shorten evacuation time and reduce casualties. From a micro perspective, after a disaster, residents do not know where to evacuate or which road to take, and they tend to follow the crowd, which will cause the number of people serving in some shelters to exceed their maximum capacity, and the possibility of stepping accident will be greatly increased. Therefore, according to the allocation results of shelters, the evacuation route of each shelter is analyzed with the help of the ArcGIS network analysis tool, as shown in Figure 12, in which the green stars represent the shelters, the light green rectangles represent the residential buildings, and the black and red lines represent the actual road network and the evacuation paths covered by the shelters, respectively. Assigning an evacuation destination to residents and helping them draw an evacuation route map can let residents know where to go and which road to take, thereby forming an orderly evacuation. From a macro perspective, the evacuation heat map is drawn according to the number of times each road is used as an evacuation route, as shown in Figure 13, we can formulate corresponding management measures according to the road color. When the road color tends to red, the road sections have the largest traffic volume, which is more prone to congestion, and the traffic flow of these road sections can be diverted to the road sections with green. The road sections with orange color have a large traffic volume, which are potential congestion areas. To avoid congestion, we can maintain the road unobstructed through traffic controllers.

## 5. Conclusion

The main contribution of this study is to solve the problem of the distribution of shelters and to make a plan of evacuation routes for each building. Appropriate shelter allocation can improve evacuation efficiency. To solve the problem of shelter allocation, we propose a mathematical model, in which three objectives and one constraint are considered, which are to minimize the evacuation distance F1, to minimize the discrete distance F2, to maximize the combined rationality F3, and to meet the capacity constraint, so as to describe the problem of shelter allocation.

In this study, we propose an integrated method combining spreading operation and a quantum genetic algorithm to solve the problem of shelter allocation. To improve the efficiency and reduce the complexity of the analysis, it is converted from the evacuation demand point as an analytical variable to the evacuation site as an analytical variable. In each iteration, the spreading operation is used to calculate the fitness of the improved quantum genetic algorithm, and the shelter allocation scheme is gradually generated. In addition, we also analyze the rationality of the shelter allocation scheme in different scenarios. The results show that the QGA generated by spreading operation is more reasonable and the optimization efficiency is better. Finally, we analyze the evacuation route of each shelter according to the allocation scheme and then calculate the number of times that each path is the shortest path in the evacuation process; as a result, we have drawn the evacuation heat map.

In this study, we assume that every road is reliable optimization, but after a disaster, the road is more likely to be damaged. Therefore, we need to consider the probability of road damage in future work. In addition, we should consider the influence of congestion, safety, fairness, and other factors in the evacuation process in order to build a multi-objective optimization function. Furthermore, the disasters faced by humans are often not single, but diverse or even simultaneous. Therefore, shelters against comprehensive disasters are important and necessary. In urban disaster prevention planning, the design and construction of comprehensive shelters need more in-depth discussion.

## Data availability statement

The original contributions presented in the study are included in the article/supplementary material, further inquiries can be directed to the corresponding author.

## Author contributions

WL: funding acquisition, revision discussion, and review and editing. YY and XZ: conceptualization and writing—original draft. YY: formal analysis, methodology, and revising. All authors contributed to the article and approved the submitted version.
